# Relationship between lncRNA-Ang362 and prognosis of patients with coronary heart disease after percutaneous coronary intervention

**DOI:** 10.1042/BSR20201524

**Published:** 2020-07-24

**Authors:** Hui Wang, Huichao Gong, Yingwu Liu, Limin Feng

**Affiliations:** 1 Heart Center, Third Central Hospital of Tianjin, Tianjin, China; 2 Tianjin Institute of Hepatobiliary Disease, Tianjin, China; 3 Tianjin Key Laboratory of Artificial Cell, Tianjin, China; 4 Artificial Cell Engineering Technology Research Center of Public Health Ministry, Tianjin, China; 5 Traditional Chinese Medicine Department, Liulin Hospital of Tianjin Hexi District; 6 Department of Classic Traditional Chinese Medicine, The Second Affiliated Hospital of Tianjin University of Traditional Chinese Medicine, Tianjin City, China

**Keywords:** cohort study, Coronary heart disease, lncRNA, percutaneous coronary intervention

## Abstract

The severity and complexity evaluation of coronary artery disease in patients with coronary heart disease (CHD) require objective and accurate prognosis indexes. We assessed the relationship between lncRNA-Ang362 and prognosis of CHD patients after percutaneous coronary intervention (PCI). Clinical follow-up data of CHD patients were prospectively collected. LncRNA-Ang362 levels were detected by real-time quantitative polymerase chain reaction. Survival rate was calculated by the Kaplan–Meier method, and risk ratios and 95% confidence intervals were computed using univariate and multivariate COX proportional hazard models. Finally, 434 patients were included in the follow-up cohort. The median follow-up time was 24.8 months (6.7–40). The incidence of adverse cardiovascular events was 13.6%. The high expression group significantly tended to be smoker and higher body mass index, low-density lipoprotein cholesterol, high-sensitivity C-reactive protein, creatinine, and uric acid levels compared with the low expression group. According to the SYNTAX grade, the high-risk and medium-risk groups had significantly higher lncRNA expression levels than the low-risk group. The univariate COX regression analysis indicated that high lncRAN-Ang362 expression significantly increased the risk of adverse cardiovascular events in CHD patients after PCI (hazard risk (HR) = 3.19, 95% confidence interval (CI): 1.29–7.92). Multivariate analysis found high lncRNA-Ang362 expression was independently related to worse prognosis in CHD patients after PCI (HR = 2.83, 95%CI: 1.34–6.02). Plasma lncRNA-Ang362 may be a prognosis factor in CHD patients after PCI. The patients with higher lncRNA-Ang362 expression usually have poor prognosis.

## Introduction

Coronary atherosclerotic heart disease, often referred to as coronary heart disease (CHD), is a heart disease caused by coronary artery stenosis or occlusion, resulting in myocardial ischemia, hypoxia or necrosis [1]. CHD is a middle-aged and elderly disease, since it commonly attacks adults over 40 years old. The incidence of CHD is gradually equal to that in men [2]. However, CHD is becoming more prevalent among younger people in recent years. The morbidity and mortality of CHD are in the first place of chronic non-communicable diseases, which seriously threaten human health and place a huge economy burden for patients and the society [3]. CHD is caused by progressive coronary atherosclerosis and influenced by many factors, including age, gender, obesity, smoking, hyperglycemia, hyperlipidemia, hypertension, hyper-homocysteine, hyper-uric acid, procalcitonin, platelet to lymphocyte, and genetic factors [4–6]. Revascularization is the most effective treatment for CHD. Although it cannot completely cure CHD, it can greatly improve the clinical symptoms of CHD patients and raise their life quality and survival rate [7]. At present, coronary artery revascularization mainly includes coronary artery bypass grafting (CABG) and percutaneous coronary intervention (PCI) [8]. With the advent of PCI, its rapid development and the continuous emergence of new anticoagulant and antiplatelet drugs has continuously broken through the restricted area of PCI treatment [9]. Nevertheless, the severity and complexity evaluation of coronary artery disease (CAD) in CHD patients require objective and accurate indexes.

Long non-coding RNA (lncRNA), a new type of non-coding RNA, is more than 200 nucleotides in length and does not encode proteins [10]. The lncRNA expression in the heart or circulation of patients with myocardial infarction may be significantly abnormal [11,12]. As reported, lncRNA-LIPCAR can predict heart failure after myocardial infarction, suggesting that lncRNA is expected to be a new molecular marker of heart disease [13]. LncRNA-Ang362 can up-regulate angiotensin II and is located between miR-222 and miR-221, which both can interact with angiotensin II and regulate the proliferation of vascular smooth muscle cells (VSMCs) [14]. The similar regulating mechanism indicates that lncRNA-Ang362 may impact the development of atherosclerosis through regulating VSMC proliferation. The present study hypothesized that lncRNA-Ang362 may be released into circulation during the procession of CHD. We explored the changes of plasma lncRNA-Ang362 expression during the development of CHD and the feasibility of using lncRNA-Ang362 as a potential prognosis biomarker for CHD after PCI.

## Materials and methods

### Study population

We enrolled CHD patients who received coronary arteriography in the Second Affiliated Hospital of Tianjin University of Traditional Chinese Medicine between June 2011 and July 2014. CHD diagnosed by referring to the American College of Cardiology Coronary Scoring [15]. All patients were confirmed by two experienced clinicians. Criteria for inclusion were: stable angina pectoris, unstable angina pectoris or acute myocardial infarction with ischemic evidences (including electrocardiograph, treadmill test, coronary computed tomography angiography or radionuclide myocardial perfusion imaging), interfered stenotic plaque, vessel diameter ≥1.5 mm and stenosis ≥70% by angiography. Criteria for exclusion were: history of PCI, coronary artery bypass grafting (CABG) and valve replacement valvular dyslipidemia, severe congenital dyslipidemia, dilated desmopathy, severe pulmonary hyper derma and ventricular septal perforation, severe liver, kidney and lung diseases, definite bacterial and fungal infections, severe anemia, severe malnutrition and terminal malignant tumor, unmedicated rheumatic diseases, hyperthyroidism, and hypothyroidism. The present study was approved by the ethics committee of The Second Affiliated Hospital of Tianjin University of Traditional Chinese Medicine. The research has been carried out in accordance with the World Medical Association Declaration of Helsinki, and that all subjects provided informed consent.

### Clinical data collection and relevant definition

Clinical data were collected by questionnaire. The data source was from medical records. From each subject, the following data were collected: age (year), gender (male vs. female), history of diabetes, hypertension (systolic blood pressure≥140 mmHg and/or diastolic blood pressure≥90 mmHg) [16], smoking (at least five cigarettes a day for more than one year), drinking (2–4 times a month) [17], body mass index (BMI = body weight/height^2^ kg/m^2^; normal: 18.5–24; overweight: 24–28; obesity:≥28) [18]. All patients were graded on SYNTAX (www.syntaxscore.com), and the CAD patients were again divided into three groups: a low-risk group (SYNTAX score<23), a medium-risk group (SYNTAX score 23-32) and a high-risk group (SYNTAX score>32) [19]. The biochemical parameters were detected by a biochemical analyzer (Hitachi, Japan), including blood glucose, total cholesterol (TC), triglyceride (TG), low-density lipoprotein cholesterol (LDL-C), high-density lipoprotein cholesterol (HDL-C), high-sensitivity C-reactive protein (hs-CRP), creatinine, and uric acid. The type of CHD, lesion count, and left ventricular ejection fraction (LVEF) were collected by medical records.

### Plasma lncRNA level

Fasting blood samples were collected before coronary angiography from each subject. In the morning, ethylenediaminetetraacetic acid dipotassium salt was used for anticoagulation. The samples were centrifuged at 3000 r/min and 4°C for 15 min and were immediately separated to collect plasma. LncRNA-Ang362 levels were detected by real-time quantitative polymerase chain reaction (qRT-PCR) as follows: total RNA from the plasma to be tested was extracted using a TRIzol reagent, and isopropanol was replaced by 75% ethanol for RNA precipitation. RNA quality was measured using a NanoDrop 1000 spectrophotometer. A total of 1 mµ gRNA was reversed into cDNA using a DBI Bestar qPCR RT kit (DBI Bioscience, Ludwigshafen, Germany). The qRT-PCR conditions performed on a 7500 Fast real-time PCR instrument were: incubation at 95°C for 2 min, and then annealing at 95°C for 15 s and 60 s for 40 cycles. LncRNA-Ang362 PCR primers were designed by Raybo Biotechnology Co., Ltd. The F and R sequences were as follows: 5′ -TGAGGGCAAGATGACAAAGA-3′, 5′-GCCCTTGCACTTGATGGTAT-3′ (lnc-Ang362); 5′-CTCGCTTCGGCAGCACAT ATACT-3′, 5′-ACGCTTCACGAATTTGCGTGTC-3′ (U6 as internal reference). MicroRNAs were quantitatively analyzed by the 2^−ΔΔCt^ method. The process was repeated three times and the average value was used.

### Follow-up

Regular telephone follow-up or outpatient follow-up after discharge was conducted at least once a month to record major adverse cardiovascular events of the patients after discharge, including death, nonfatal myocardial infarction, and revascularization (from the post-operation to occurrence of events). Myocardial infarction was defined as chest pain persisting for more than 20 min or abnormal changes of electrocardiogram accompanied by increased troponin anomaly. Revascularization was defined as retreatment of previous diseased vessels by surgery or interventional surgery or the treatment of new vascular stenosis.

### Statistical analysis

Statistical analysis was performed on SPSS 20.0 and Graphpad 8.0, and normality test was carried out for quantitative data first. The study population was divided into a high expression group and a low expression group according to the median lncRN-Ang362 level. Data with normal distribution were expressed as mean ± standard deviation, and compared between groups by two independent sample *t*-test. Data of skewness distribution were represented by median, and compared between groups through nonparametric rank sum test. Categorical data were expressed by counts and percent and compared between groups via the Chi-square test. Survival rate was calculated using the Kaplan–Meier method, and risk ratio (HR) and 95% confidence intervals (CIs) were computed through univariate and multivariate COX proportional hazard models. The adjusted factors for multivariate analysis included age, gender, smoking, drinking, hypertension, diabetes mellitus, BMI, TC, TG, LDL-C, HDL-C, hs-CRP, creatinine, uric acid, vessel lesions, and LVEF. *P*<0.05 was considered as statistically significant.

## Results

### General characteristics

Initially, we obtained data from 612 CHD patients who received PCI. According to the inclusion and exclusion criteria, we excluded 178 patients owing to the history of CABG (*n*=22), history of PCI (*n*=56), valve replacement (*n*=13), severe congenital dyslipidemia (*n*=12), dilated desmopathy (*n*=9), tumor (*n*=12), severe liver and kidney dysfunction (*n*=23), and incomplete follow-up data (*n*=31). Finally, 434 patients including 218 males and 216 females were included in the follow-up cohort. Their mean age was 66.0 ± 5.9 years old. The ratios of hypertension and diabetes were 29.0% (*n*=126) and 26.5%, respectively. The ratios of history of smoking and drinking were 36.6% (*n*=159) and 30.0% (*n*=130), respectively. The ratios of acute myocardial infraction, unstable angina pectoris and ischemic cardiomyopathy were 35.7%, 39.9%, and 24.4%, respectively. About 82.3% of patients had more than one lesion vessel. The median follow-up time was 24.8 months (6.7–40.0). After more than 3-year follow-up, 59 (13.6%) patients had adverse cardiovascular events, including 6 deaths, 21 patients with revascularization and 22 patients with myocardial infarctions.

### LncRNa-Ang362 and clinical parameters

**Figure 1:**
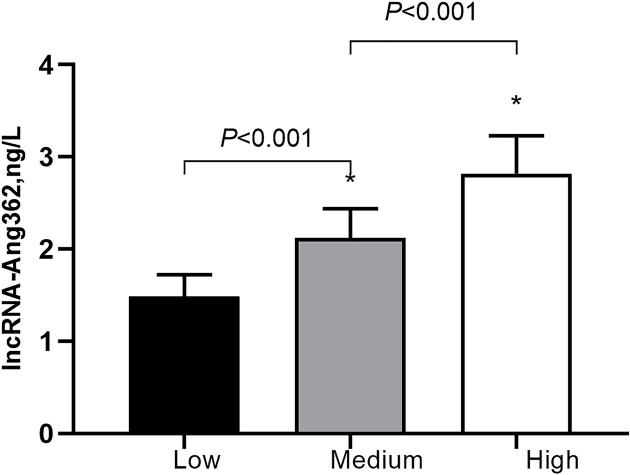
Histogram of lncRNA-Ang362 expression Relationship between lncRNA-Ang362 expression and SYNTAX grade (high vs. low: *P*<0.05; Medium vs. low: *P*<0.05; High vs. Medium: *P*<0.05). *P<0.05.

The study population was divided by the median lncRAN-Ang362 level into a high expression group (*n*=217) and a low expression group (*n*=217). The high expression group versus the low expression group tended to be smokers (50.8% vs. 45.8%, *P*=0.024) and had higher BMI (27.2 ± 3.2 vs 24.2 ± 3.3, *P*<0.001), LDL-C (3.1 ± 0.8 vs 2.6 ± 0.7, *P*<0.001), hs-CRP, creatinine (7.8 ± 1.7 vs. 5.2 ± 0.6, *P*<0.001), and uric acid levels (371.4 ± 79.1 vs. 327.4 ± 68.2, *P*<0.001), but significantly smaller LVEF (53.1 ± 8.9 vs. 58.7 ± 7.2, *P*<0.001). We compared the lncRNA-Ang362 levels among different SYNTAX grades and found the high-risk and medium-risk groups had significantly higher lncRNA expression levels than the low-risk group (*P*<0.05; Figure 1). No significant differences were observed in age (*P*=0.098), male ratio (*P*=0.701), hypertension ratio (*P*=0.057), diabetes ratio (*P*=0.328), drinking ratio (*P*=0.209), or several biochemical parameters, including blood glucose level (*P*=0.565), TC (*P*=0.547), TG (*P*=0.388), and HDL-C (*P*=0.898). The ratios of acute myocardial infarction, unstable angina pectoris and ischemic cardiomyopathy were 33.2%, 35.5%, and 21.7%, respectively, in the low expression group and 38.32%, 44.2%, and 27.2%, respectively, in the high expression group, without significant differences between groups (*P*=0.270, 0.063, and 0.180, respectively). We also did not observe significant difference in the lesion count of vessel (*P*=0.102). The detailed information is presented in Table 1.

**Table 1:** Baseline demographics of study population by lncRNA-Ang362

Factors	Low expression (*n*=217)	High expression (*n*=217)	χ^2^/t	*P*
Age (year)	65.7 ± 5.8	66.3 ± 6.1	1.043	0.298
Male (*n*, %)	107 (49.3%)	111 (51.2%)	0.147	0.701
Hypertension (*n*, %)	54 (24.9%)	72 (33.2%)	3.623	0.057
Diabetes (*n*, %)	53 (24.4%)	62 (28.6%)	0.958	0.328
Smoking (*n*, %)	96 (45.8%)	63 (50.8%)	5.104	0.024
Drinking (*n*, %)	59 (27.2%)	71 (32.7%)	1.581	0.209
BMI, kg/m^2^	24.2 ± 3.3	27.2 ± 3.2	9.614	0.000
Blood glucose, mmol/l	6.3 ± 1.6	6.4 ± 2.0	0.575	0.565
TC, mmol/l	4.5 ± 2.0	4.6 ± 1.4	0.603	0.547
TG, mmol/l	1.6 ± 1.3	1.7 ± 1.1	0.865	0.388
LDL-C, mmol/l	2.6 ± 0.7	3.1 ± 0.8	6.929	0.000
HDL-C, mmol/l	1.1 ± 0.2	1.0 ± 0.2	0.128	0.898
Hs-CRP, mg/l	5.2 ± 0.6	7.8 ± 1.7	21.245	0.000
Creatinine, mmol/l	64 (52–76)	75 (62–86)	14.78	0.000
Uric acid, mmol/l	327.4 ± 68.2	371.4 ± 79.1	6.206	0.000
AMI (*n*, %)	72 (33.2%)	83 (38.2%)	1.214	0.270
UAP (*n*, %)	77 (35.5%)	96 (44.2%)	3.470	0.063
Ischemic cardiomyopathy	47 (21.7%)	59 (27.2%)	1.797	0.180
Lesion count			2.668	0.102
Single vessel (*n*, %)	32 (14.7%)	45 (20.7%)	6.201	
Multiple vessel (*n*, %)	185 (85.3%)	172 (79.3%%)	1.657	
LVEF (%)	58.7 ± 7.2	53.1 ± 8.9	7.206	0.000

Abbreviations: AMI, acute myocardial infarction; BMI, body mass index; EATV, epicardial adipose tissue volume; HDL, high density lipoprotein; Hs-CRP, high-sensitivity C-reactive protein; LDL, low density lipoprotein; LVEF, left ventricular ejection fraction; TC, total cholesterol; TG, triglyceride; UAP, unstable angina pectoris.

χ^2^: Chi-square test; *t*: two independent *t* test.

### LncRNa-Ang362 and prognosis of CHD after PCI

**Figure 2:**
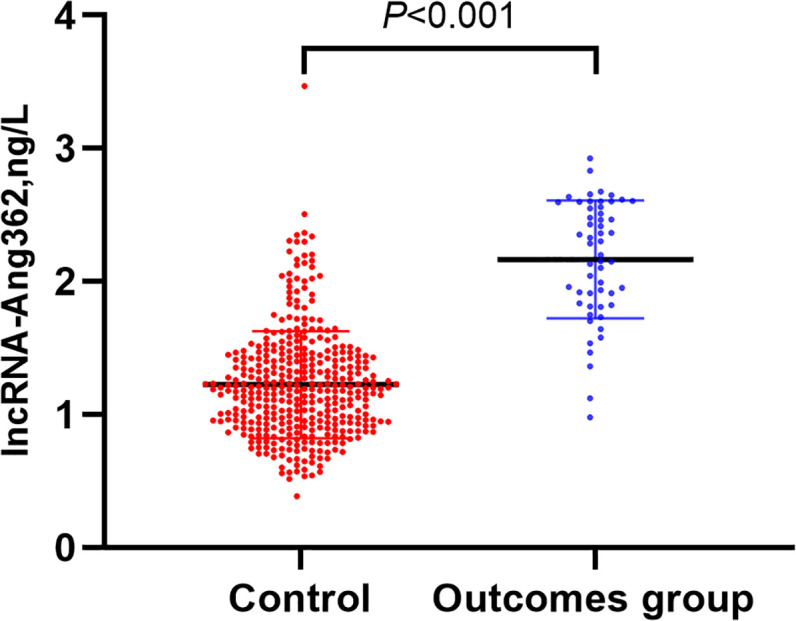
Comparison of lncRNA-Ang362 expression between outcomes and control group

**Figure 3:**
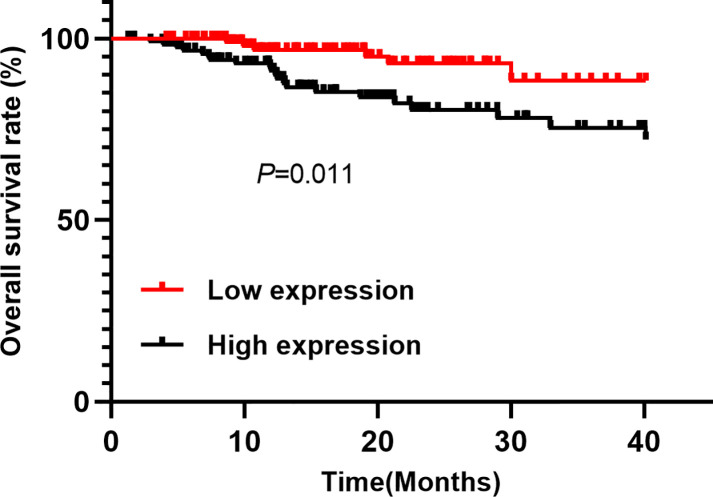
Kaplan–Meier survival curve of lncRNA-Ang362 high expression and low expression group

These patients with follow-up outcomes (major adverse cardiovascular events) group had higher lncRNA expression level than that without cardiovascular events (Figure 2). The univariate COX regression analysis (Table 2) indicated that high lncRAN-Ang362 expression increased the risk of adverse cardiovascular events in CHD patients after PCI (HR = 3.19, 95%CI: 1.29–7.92, *P*<0.001). Poor prognosis in CHD patients after PCI was positively associated with smoking (HR = 2.65, 95%CI: 1.61–3.67, *P*<0.001), overweight/obesity (1.66, 1.25–3.29, *P*<0.001), high LDL-C (1.85, 1.31–2.76, *P*<0.001), hs-CRP elevation (3.75, 1.26–4.13, *P*<0.001), elevated creatinine (1.86, 1.24–2.75, *P*<0.001), uric acid level (1.22, 1.18–2.12, *P*=0.010), and multiple vessel lesion (2.04, 1.13–6.21, *P*=0.005). The Kaplan–Meier analysis indicated the high expression group of lncRNA-Ang362 had poorer overall survival rate compared with the low expression group (*P*=0.011, Figure 3).

**Table 2:** Univariate Cox regression analysis to assess independent correlated of adverse events following PCI for CHD during follow-up

Factors	*B*	*SE*	*Waldχ^2^*	*P*	HR (95%CI)
Age ≥60	0.342	0.018	1.854	0.253	1.41 (0.87–3.15)
Male	0.118	0.385	0.871	0.432	1.13 (0.96–2.69)
Hypertension	0.426	0.396	0.862	0.501	1.53 (0.90–1.49)
Smoking	0.501	0.462	12.983	0.000	2.65 (1.61–3.67)
Drinking	0.384	0.518	0.895	0.469	1.46 (0.49–2.26)
BMI ≥ 28 kg/m^2^	0.509	0.148	15.114	0.000	1.66 (1.25–3.29)
Blood glucose ≥ 6.1 mmol/l	0.051	0.021	1.612	0.113	1.05 (0.17–1.69)
TC ≥ 5.18 mmol/l	0.563	0.793	0.533	0.665	1.52 (0.47–2.89)
TG ≥ 1.7 mmol/l	-0.287	0.858	0.212	0.865	0.75 (0.32–2.52)
LDL-C ≥ 3.37 mmol/l	0.616	0.476	20.658	0.000	1.85 (1.31–2.76)
HDL-C < 1.04 mmol/l	-0.215	0.434	0.563	0.521	0.81 (0.41–2.02)
Hs-CRP, mg/dl	1.324	0.141	14.875	0.000	3.75 (1.26–4.13)
Creatinine, μmol/l	0.224	0.112	21.512	0.000	1.86 (1.24–2.75)
Uric acid, μmol/l	0.026	0.101	9.623	0.010	1.22 (1.18–2.12)
Multiple vessel (*n*, %)	0.713	0.301	10.210	0.005	2.04 (1.13–6.21)
LVEF (%)	0.102	0.081	1.268	0.193	1.11 (0.78–2.22)
lncRNA-Ang362 (high vs. low)	1.160	0.464	16.25	0.000	3.19 (1.29–7.92)

Abbreviations: AMI, acute myocardial infarction; BMI, body mass index; EATV, epicardial adipose tissue volume; HDL, high density lipoprotein; Hs-CRP, high-sensitivity C-reactive protein; LDL, low density lipoprotein; LVEF, left ventricular ejection fraction; TC, total cholesterol; TG, triglyceride; UAP, unstable angina pectoris.

The multivariate analysis found that high lncRNA-Ang362 expression was independently related to worse prognosis in CHD patients after PCI (HR = 2.83, 95%CI: 1.34–6.02, *P*=0.007) (Table 3). Poor prognosis in CHD patients after PCI was also positively related to high BMI, LDL-C, hs-CRP, and creatinine.

**Table 3:** Multivariate COX regression analysis to assess independent correlated of adverse events following PCI for CHD during follow-up

Factors	*B*	*SE*	*Waldχ^2^*	*P*	HR (95%CI)
Smoking	0.221	0.331	11.283	0.021	1.24 (1.17–2.31)
BMI ≥ 28 kg/m^2^	0.340	0.461	10.814	0.026	1.40 (1.19–2.74)
LDL-C ≥ 3.37 mmol/l	0.523	0.236	12.369	0.011	1.69 (1.22–3.45)
Hc-CRP, mg/dl	1.197	0.393	20.124	0.000	3.31 (1.24–3.89)
Creatinine, μmol/l	0.528	0.213	11.478	0.010	1.71 (1.17–2.65)
lncRNA-Ang362 (high vs low)	1.043	0.384	13.025	0.007	2.83 (1.34–6.02)

Adjusting for potential confounding factors, including age, gender, smoking, drinking, hypertension, diabetes mellitus, BMI, TC, TG, LDL-C, HDL-C, hs-CRP, creatinine, uric acid, vessel lesions, and LVEF.

## Discussion

LncRAN-Ang362 was found to be highly expressed in CHD patients with adverse outcomes. The high expression group of lncRNA-Ang362 had better overall survival rate compared with the low expression group. The multivariate analysis indicated high lncRNA-Ang362 expression was independently associated with poor prognosis in CHD patients after PCI. Our study provided a potential prognosis biomarker of CHD after PCI.

Cardiovascular diseases, especially CHD, are still the major cause of death worldwide, causing a major socioeconomic burden. Although therapeutic methods such as medications, PCI and CABG have improved the prognosis of CHD, the mortality rate remains high [20]. Therefore, it is necessary to detect CHD in its earlier stage, especially before the development of left ventricular dysfunction. Early identification of CHD patients at high risk of adverse cardiovascular events using circulating or imaging biomarkers many help in this regard [21]. However, the lack of available CAD biomarkers has limited risk prediction [22].

Genome-wide analysis has identified that almost all of the human genome is transcribed, with a large amount of lncRNAs [23,24]. LncRNAs range over 200 nucleotides in length [25], and, through epigenetic, transcriptional, or post-transcriptional regulatory mechanisms, are involved in specific physiological and pathological processes of a wide range of human diseases and disorders [26], such as cancers [27] and neurological disorders [28]. As reported recently, some lncRNAs are involved in the development of various cardiovascular diseases [29], including heart failure [30,31], cardiac hypertrophy [32], and myocardial infarction [33]. The plasma levels of some lncRNAs, such as ANRIL, LincRNA-p21 and myocardial infarct-associated transcript-1, markedly increase in atherosclerosis and may be important in its pathogenesis. LncRNAs are stable in plasma and other body fluids and therefore, can serve as biomarkers for some diseases [34]. For example, a prostate-specific lncRNA PCA3 in urine has been identified as the most specific biomarker for detection of prostate cancer with higher specificity than the widely-used prostate- specific antigen test [35]. Other lncRNA biomarkers in plasma include the lncRNA LIPCAR for heart failure post-myocardial infarction and CHD [36–38]. Moreover, miRNA-221 and miRNA-222 are involved in VSMC proliferation and are elevated in response to Ang II in endothelial cells to promote inflammation and migration. LncRNA-Ang362 is an Ang II-upregulated lncRNA proximal to these two miRNAs, which means that lncRNA and these two miRNAs have a synergistic effect on VSMC proliferation [39]. CHD is mainly caused by atherosclerosis, in which phenotypic transformation and aggregation migration of VSMCs play an important role [40]. These findings suggest that lncRNA-Ang362 is involved in the development of CHD. Our results indicate the lncRNA-Ang362 expression is the severity of CHD and is positively associated with SYNTAX grade, suggesting lncRNA-Ang362 can be a potential prognosis biomarker. Furthermore, the high expression of lncRNA-Ang362 corresponds to a higher incidence of adverse cardiovascular events. This result can be also explained by the synergistic effect of lncRNA with miRNA-221 and miRNA-222. The minichromosomal maintenance protein (MCM) 7 is a key molecule of the lncRNA-Ang362 regulation group that promotes cell proliferation and regulates cell cycle [41]. Excessive activation of MCM7 can lead to excessive proliferation and activity of VSMCs, which often indicates accelerated atherosclerotic process and unstable atherosclerotic plaques in patients [42]. This finding may partly explain why lncRNA-Ang362 can affect the prognosis of CHD patients.

We found that high lncRNA-Ang362 expression was associated with elevated hc-CRP, a marker of inflammation status, which indicates lncRNA-Ang362 is involved in the process of inflammation response. As reported, lncRNA-Ang362 regulates the biological function of angiotensin II that is necessary in regulating VSMC proliferation via promoting inflammation response [43]. The levels of creatinine and uric acid, two kidney function markers, were also positively associated with lncRNA-Ang362. The creatinine and uric acid elevation mean a decreased kidney function, indicating lncRNA-Ang362 may be related to kidney function. Nevertheless, further research is required.

The present study has several limitations. First, this is a single-center study with limited sample size, and further subgroup analysis cannot be obtained. Second, we did not collect data of medicine treatments from some hypertensive or diabetic patients who may be taking medicine, but these treatments may affect the final estimation. Third, we did not explore the molecular mechanisms of the relationship between lnc-Ang362 and CHD severity, which should be tested by research *in vivo* and *in vitro*. Finally, the present result was based on short-term follow-up and thus must be extended by long-term follow-up data.

In conclusion, lncRNA-Ang362 is an independent prognosis biomarker of CHD patients after PCI. Our results provide evidences for the prognosis outcome improvement because lncRNA-Ang362 may be a potential treatment target for CHD after PCI. Studies with larger sample size and longer time follow-up are required to confirm our finding. Future research should also focus on the molecular mechanism.

## Abbreviations

aRT-PCR: real-time quantitative polymerase chain reaction
CABG: coronary artery bypass grafting
CAD: coronary artery disease
CHD: coronary heart disease
CI: confidence interval
HDL-C: high-density lipoprotein cholesterol
HR: hazard risk
hs-CRP: high sensitivity C-reactive protein
LDL-C: low-density lipoprotein cholesterol
LVEF: left ventricular ejection fraction
PCI: percutaneous coronary intervention
TC: total cholesterol
TG: triglyceride
VSMC: vascular smooth muscle cell

## References

[1] Sanchis-Gomar F., Perez-Quilis C., Leischik R. and Lucia A. (2016) Epidemiology of coronary heart disease and acute coronary syndrome. Ann. Transl. Med. 4, 256 10.21037/atm.2016.06.33 27500157 PMC4958723

[2] Roth G.A., Johnson C., Abajobir A., Abd-Allah F., Abera S.F., Abyu G. et al. (2017) Global, Regional, and National Burden of Cardiovascular Diseases for 10 Causes, 1990 to 2015. J. Am. Coll. Cardiol. 70, 1–25 10.1016/j.jacc.2017.04.052 28527533 PMC5491406

[3] (2018) The changing patterns of cardiovascular diseases and their risk factors in the states of India: the Global Burden of Disease Study 1990-2016. Lancet Glob. Health 6, e1339–e1351 10.1016/S2214-109X(18)30407-8 30219317 PMC6227386

[4] Bai M.F. and Wang X. (2019) Risk factors associated with coronary heart disease in women: a systematic review. Herz 2019, 10.1007/s00059-019-4835-2 31317202

[5] Kurtul A. and Elcik D. (2017) Procalcitonin is an independent predictor for coronary atherosclerotic burden in patients with stable coronary artery disease. Int. J. Cardiol. 236, 61–64 10.1016/j.ijcard.2017.02.061 28256322

[6] Kurtul A., Murat S.N., Yarlioglues M., Duran M., Ergun G., Acikgoz S.K. et al. (2014) Association of platelet-to-lymphocyte ratio with severity and complexity of coronary artery disease in patients with acute coronary syndromes. Am. J. Cardiol. 114, 972–978 10.1016/j.amjcard.2014.07.005 25118117

[7] Berry C., Tardif J.C. and Bourassa M.G. (2007) Coronary heart disease in patients with diabetes: part II: recent advances in coronary revascularization. J. Am. Coll. Cardiol. 49, 643–656 10.1016/j.jacc.2006.09.045 17291929

[8] Kiaii B. and Teefy P. (2019) Hybrid Coronary Artery Revascularization: A Review and Current Evidence. Innovations (Phila.) 14, 394–404 10.1177/1556984519872998 31500492

[9] Changjiang H., Jian Q., Yuan Z., Liang Y., Puqing L. and Xiaolong G. (2015) Tirofiban Combined with Fondaparinux for Post-PCI Treatment of Patients with Acute Coronary Syndrome and Mild Renal Insufficiency. Cell Biochem. Biophys. 73, 603–607 10.1007/s12013-015-0580-1 27259300

[10] Bunch H. (2018) Gene regulation of mammalian long non-coding RNA. Mol. Genet. Genomics 293, 1–15 10.1007/s00438-017-1370-9 28894972

[11] Bink D.I., Lozano-Vidal N. and Boon R.A. (2019) Long Non-Coding RNA in Vascular Disease and Aging. Noncoding RNA 5, 30893946 10.3390/ncrna5010026 PMC6468806

[12] Cai Y., Yang Y., Chen X., He D., Zhang X., Wen X. et al. (2016) Circulating “LncPPARdelta” From Monocytes as a Novel Biomarker for Coronary Artery Diseases. Medicine (Baltimore) 95, e2360 10.1097/MD.0000000000002360 26871769 PMC4753863

[13] Zhang Z., Gao W., Long Q.Q., Zhang J., Li Y.F., Liu D.C. et al. (2017) Increased plasma levels of lncRNA H19 and LIPCAR are associated with increased risk of coronary artery disease in a Chinese population. Sci. Rep. 7, 7491 10.1038/s41598-017-07611-z 28790415 PMC5548926

[14] Leung A., Trac C., Jin W., Lanting L., Akbany A., Saetrom P. et al. (2013) Novel long noncoding RNAs are regulated by angiotensin II in vascular smooth muscle cells. Circ. Res. 113, 266–278 10.1161/CIRCRESAHA.112.300849 23697773 PMC3763837

[15] Fihn S.D., Gardin J.M., Abrams J., Berra K., Blankenship J.C., Dallas A.P. et al. (2012) 2012 ACCF/AHA/ACP/AATS/PCNA/SCAI/STS guideline for the diagnosis and management of patients with stable ischemic heart disease: executive summary: a report of the American College of Cardiology Foundation/American Heart Association task force on practice guidelines, and the American College of Physicians, American Association for Thoracic Surgery, Preventive Cardiovascular Nurses Association, Society for Cardiovascular Angiography and Interventions, and Society of Thoracic Surgeons. Circulation 126, 3097–3137 23166210 10.1161/CIR.0b013e3182776f83

[16] Xu F. and Shen L. (2019) Diagnosis and treatment of hypertension in the Republic of China. Zhonghua Yi Shi Za Zhi 49, 79–82 31137155 10.3760/cma.j.issn.0255-7053.2019.02.003

[17] Lu C., Jia H. and Wang Z. (2019) Association between epicardial adipose tissue and adverse outcomes in coronary heart disease patients with percutaneous coronary intervention. Biosci. Rep. 39, 10.1042/BSR20182278 PMC6504663 30979830

[18] Wang Y., Xue H., Sun M., Zhu X., Zhao L. and Yang Y. (2019) Prevention and control of obesity in China. Lancet Glob. Health 7, e1166–e1167 10.1016/S2214-109X(19)30276-1 31401995

[19] Modolo R., Collet C., Onuma Y. and Serruys P.W. (2018) SYNTAX II and SYNTAX III trials: what is the take home message for surgeons? Ann. Cardiothorac. Surg. 7, 470–482 10.21037/acs.2018.07.02 30094211 PMC6082789

[20] Daneault B., Genereux P., Kirtane A.J., Witzenbichler B., Guagliumi G., Paradis J.M. et al. (2013) Comparison of Three-year outcomes after primary percutaneous coronary intervention in patients with left ventricular ejection fraction &lt;40% versus >/ = 40% (from the HORIZONS-AMI trial). Am. J. Cardiol. 111, 12–20 23040595 10.1016/j.amjcard.2012.08.040

[21] Malaud E., Merle D., Piquer D., Molina L., Salvetat N., Rubrecht L. et al. (2014) Local carotid atherosclerotic plaque proteins for the identification of circulating biomarkers in coronary patients. Atherosclerosis 233, 551–558 10.1016/j.atherosclerosis.2013.12.019 24530963

[22] Wykrzykowska J.J., Garcia-Garcia H.M., Goedhart D., Zalewski A. and Serruys P.W. (2011) Differential protein biomarker expression and their time-course in patients with a spectrum of stable and unstable coronary syndromes in the Integrated Biomarker and Imaging Study-1 (IBIS-1). Int. J. Cardiol. 149, 10–16 10.1016/j.ijcard.2009.11.033 20060182

[23] Guttman M., Amit I., Garber M., French C., Lin M.F., Feldser D. et al. (2009) Chromatin signature reveals over a thousand highly conserved large non-coding RNAs in mammals. Nature 458, 223–227 10.1038/nature07672 19182780 PMC2754849

[24] Ponting C.P., Oliver P.L. and Reik W. (2009) Evolution and functions of long noncoding RNAs. Cell 136, 629–641 10.1016/j.cell.2009.02.006 19239885

[25] Bertone P., Stolc V., Royce T.E., Rozowsky J.S., Urban A.E., Zhu X. et al. (2004) Global identification of human transcribed sequences with genome tiling arrays. Science 306, 2242–2246 10.1126/science.1103388 15539566

[26] Hung T., Wang Y., Lin M.F., Koegel A.K., Kotake Y., Grant G.D. et al. (2011) Extensive and coordinated transcription of noncoding RNAs within cell-cycle promoters. Nat. Genet. 43, 621–629 10.1038/ng.848 21642992 PMC3652667

[27] Weng W., Li H. and Goel A. (2019) Piwi-interacting RNAs (piRNAs) and cancer: Emerging biological concepts and potential clinical implications. Biochim. Biophys. Acta Rev. Cancer 1871, 160–169 30599187 10.1016/j.bbcan.2018.12.005 PMC6392428

[28] Niland C.N., Merry C.R. and Khalil A.M. (2012) Emerging Roles for Long Non-Coding RNAs in Cancer and Neurological Disorders. Front. Genet. 3, 25 10.3389/fgene.2012.00025 22375145 PMC3286759

[29] Schonrock N., Harvey R.P. and Mattick J.S. (2012) Long noncoding RNAs in cardiac development and pathophysiology. Circ. Res. 111, 1349–1362 10.1161/CIRCRESAHA.112.268953 23104877

[30] McKinsey T.A., Vondriska T.M. and Wang Y. (2018) Epigenomic regulation of heart failure: integrating histone marks, long noncoding RNAs, and chromatin architecture. F1000 Faculty Rev. 7, 1713 10.12688/f1000research.15797.1 30416708 PMC6206605

[31] Han D., Gao Q. and Cao F. (2017) Long noncoding RNAs (LncRNAs) - The dawning of a new treatment for cardiac hypertrophy and heart failure. Biochim. Biophys. Acta Mol. Basis Dis. 1863, 2078–2084 28259753 10.1016/j.bbadis.2017.02.024

[32] Li X., Zhang L. and Liang J. (2016) Unraveling the Expression Profiles of Long Noncoding RNAs in Rat Cardiac Hypertrophy and Functions of lncRNA BC088254 in Cardiac Hypertrophy Induced by Transverse Aortic Constriction. Cardiology 134, 84–98 10.1159/000443370 26919297

[33] Zhao P., Wu H., Zhong Z., Zhang Q., Zhong W., Li B. et al. (2018) Expression profiles of long noncoding RNAs and mRNAs in peripheral blood mononuclear cells of patients with acute myocardial infarction. Medicine (Baltimore). 97, e12604 10.1097/MD.0000000000012604 30313048 PMC6203524

[34] Rao A., Rajkumar T. and Mani S. (2017) Perspectives of long non-coding RNAs in cancer. Mol. Biol. Rep. 44, 203–218 10.1007/s11033-017-4103-6 28391434

[35] de Kok J.B., Verhaegh G.W., Roelofs R.W., Hessels D., Kiemeney L.A., Aalders T.W. et al. (2002) DD3(PCA3), a very sensitive and specific marker to detect prostate tumors. Cancer Res. 62, 2695–2698 11980670

[36] Zhang Z., Gao W., Long Q.Q., Zhang J., Li Y.F., Liu D.C. et al. (2017) Increased plasma levels of lncRNA H19 and LIPCAR are associated with increased risk of coronary artery disease in a Chinese population. Sci. Rep. 7, 7491 10.1038/s41598-017-07611-z 28790415 PMC5548926

[37] Santer L., Lopez B., Ravassa S., Baer C., Riedel I., Chatterjee S. et al. (2019) Circulating Long Noncoding RNA LIPCAR Predicts Heart Failure Outcomes in Patients Without Chronic Kidney Disease. Hypertension 73, 820–828 10.1161/HYPERTENSIONAHA.118.12261 30686085

[38] Li M., Wang Y.F., Yang X.C., Xu L., Li W.M., Xia K. et al. (2018) Circulating Long Noncoding RNA LIPCAR Acts as a Novel Biomarker in Patients with ST-Segment Elevation Myocardial Infarction. Med. Sci. Monit. 24, 5064–5070 10.12659/MSM.909348 30030914 PMC6067052

[39] Wang H., Qin R. and Cheng Y. (2020) LncRNA-Ang362 Promotes Pulmonary Arterial Hypertension by Regulating miR-221 and miR-222. Shock 53, 723–729 10.1097/SHK.0000000000001410 31313741

[40] Qiu J., Zheng Y., Hu J., Liao D., Gregersen H., Deng X. et al. (2014) Biomechanical regulation of vascular smooth muscle cell functions: from in vitro to in vivo understanding. J. R. Soc. Interface 11, 20130852 10.1098/rsif.2013.0852 24152813 PMC3836323

[41] Leung A., Trac C., Jin W., Lanting L., Akbany A., Saetrom P. et al. (2013) Novel long noncoding RNAs are regulated by angiotensin II in vascular smooth muscle cells. Circ. Res. 113, 266–278 10.1161/CIRCRESAHA.112.300849 23697773 PMC3763837

[42] Li H., Zhu H. and Ge J. (2016) Long Noncoding RNA: Recent Updates in Atherosclerosis. Int. J. Biol. Sci. 12, 898–910 10.7150/ijbs.14430 27314829 PMC4910607

[43] Adhikari N., Basi D.L., Carlson M., Mariash A., Hong Z., Lehman U. et al. (2011) Increase in GLUT1 in smooth muscle alters vascular contractility and increases inflammation in response to vascular injury. Arterioscler. Thromb. Vasc. Biol. 31, 86–94 10.1161/ATVBAHA.110.215004 20947823 PMC3014530

